# A Case Series Demonstrating the Difficulties in Diagnosing COVID-19 Associated Pulmonary Aspergillus

**DOI:** 10.7759/cureus.33802

**Published:** 2023-01-15

**Authors:** Sindhubarathi Murali, Hanish Jain, Saketh Velepati, Muhammad Alvi, Ioana Amzuta

**Affiliations:** 1 Internal Medicine, Upstate University Hospital, Syracuse, USA; 2 Pulmonary and Critical Care Medicine, Upstate University Hospital, Syracuse, USA

**Keywords:** missed diagnosis, prolonged mechanical ventilation, aspergillus bronchopneumonia, medical intensive care unit, covid-19

## Abstract

Many bacterial, viral, and fungal co-infections have been reported with COVID-19-associated acute respiratory distress syndrome (ARDS). Invasive Aspergillosis has been described with COVID-19 ARDS. However, it continues to evade diagnosis in critically ill patients admitted to the intensive care unit (ICU). The difficulty is discerning an actual infection from colonization. Unfortunately, a timely diagnosis is crucial since COVID-19-associated pulmonary Aspergillus (CAPA) has high morbidity and mortality. We present three ICU cases of CAPA to illustrate the difficulty in diagnosing and treating the disease. We hope to bring awareness and improve patient outcomes of CAPA.

## Introduction

Severe COVID-19 infection in ventilated patients with acute respiratory distress syndrome (ARDS) has been associated with invasive pulmonary aspergillus infections [1]. The structural damage of the lung architecture and the treatment of COVID-19 with steroids and immunosuppressants facilitate this co-infection. In general, those with host risk factors such as neutropenia, HIV, solid organ transplant, and chronic obstructive pulmonary disease (COPD) are more likely to develop Covid-19 Associated Pulmonary Aspergillus (CAPA) [2]. Despite the recent description of CAPA, it continues to be a difficult diagnosis with detrimental consequences in patient outcomes. The similarity in radiological signs between COVID-19 and Aspergillus is one reason for the difficulty in diagnosis [3]. The other reason is an inadequate respiratory sampling from the lower respiratory airway that would require bronchoscopy [4]. We describe three intensive care unit (ICU) cases to shed light on the early diagnosis and treatment of CAPA.

## Case presentation

Case 1

A 61-year-old female with a past medical history of depression presented to our hospital with dyspnea, fatigue, fever, vomiting, and dizziness for the past few days. She had tested positive for COVID-19 ten days prior and had not been vaccinated for COVID-19. The patient required high-flow oxygen (FiO_2_ = 100%; 60 L) and non-invasive ventilation with Bilevel Positive Airway Pressure (BIPAP; IPAP = 18, EPAP = 14). Her initial thorax computed angiography showed predominantly ground-glass opacities in the apices with atelectasis in the bases. She was started on barcitinib (PO 4 mg q24), remdesivir (initial dose IV 200 mg; IV 100 mg q24 after that for a total of 5 days), and dexamethasone (IV 6 mg q24 for a total of 10 days). However, given her worsening oxygen requirements and poor improvement, barcitinib was discontinued, and she was given a dose of sarilumab (IV 400 mg once) the following day.

On day 10, the patient was started on broad-spectrum antibiotics, vancomycin, and piperacillin-tazobactam, due to an up-trending leukocytosis. The next day, her physical exam displayed a respiratory rate of 28 breaths/min, pulse = 102, and low oxygen saturation (SpO_2_ = 84%) on BIPAP (IPAP = 18 and EPAP = 16). Arterial blood gas on a fraction of inspired oxygen (FIO_2_) of 100% demonstrated pH 7.45, partial pressure of carbon dioxide (PCO_2_) of 54 mm Hg, and partial pressure of oxygen (PaO_2_) of 82 mm Hg (PaO_2_/FiO_2_ = 82) suggestive of severe acute respiratory distress syndrome (ARDS). Elective endotracheal intubation was performed with lung protective mechanical ventilation with ventilatory settings of tidal volume (TV) 4-6 ml/kg, respiratory rate (RR) = 30, Tidal Volume (Vt) = 350, positive expiratory end pressure (PEEP) = 12, FIO_2_ = 90% with the aim of PaO_2_ > 60 mm Hg and pH > 7.2 as per ARDSnet protocol. The patient was prone multiple times to assist with alveolar recruitment. Vancomycin was discontinued once the MRSA nares were negative three days later. The patient was treated with a 7-day course of piperacillin-tazobactam. On day 20, the patient's PaO_2_/FiO_2_ (P/F) ratio improved to 241.

On day+21, an increase in temperature to 38ºC was observed. She was pan-cultured and restarted on vancomycin and meropenem. Antibiotics were discontinued once blood culture and sputum cultures were negative. Despite a negative infectious work-up, she continued to have fevers (T = 38ºC to 38.4ºC) for two weeks. On day+29, the patient suffered a bradycardia episode leading to PEA arrest. She received one round of cardiopulmonary resuscitation (CPR) and epinephrine and achieved a return of spontaneous circulation (ROSC).

**Figure 1:**
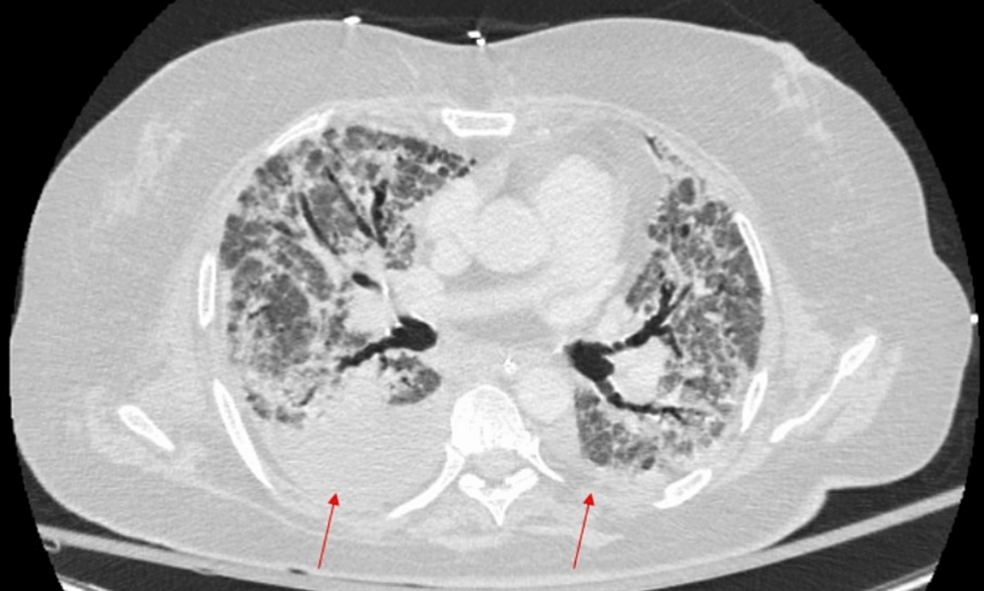
CT thorax with contrast showing lung fibrosis and moderate right and small left pleural effusion (red arrows).

Given that the patient continued to have a low-grade fever, she was pan-cultured again on Day+30. Urine culture grew Enterococcus faecalis, so she was treated with a 7-day course of piperacillin-tazobactam. Despite antibiotic treatment, the patient continued to have a low-grade fever and increasing leukocytosis (16.8 x 10^3/uL). CT thorax with contrast showed diffuse ground-glass and consolidative opacities throughout the lungs with moderate right-sided pleural and small left-sided pleural effusion (Figure 1).

Sputum Culture eventually grew 4 colonies of Aspergillus fumigatus. The Department of Infectious Disease was consulted and recommended IV voriconazole (6 mg/kg q12) for 2 doses and then transitioned to IV voriconazole (4 mg/kg q12) after that for 12 weeks.

**Figure 2:**
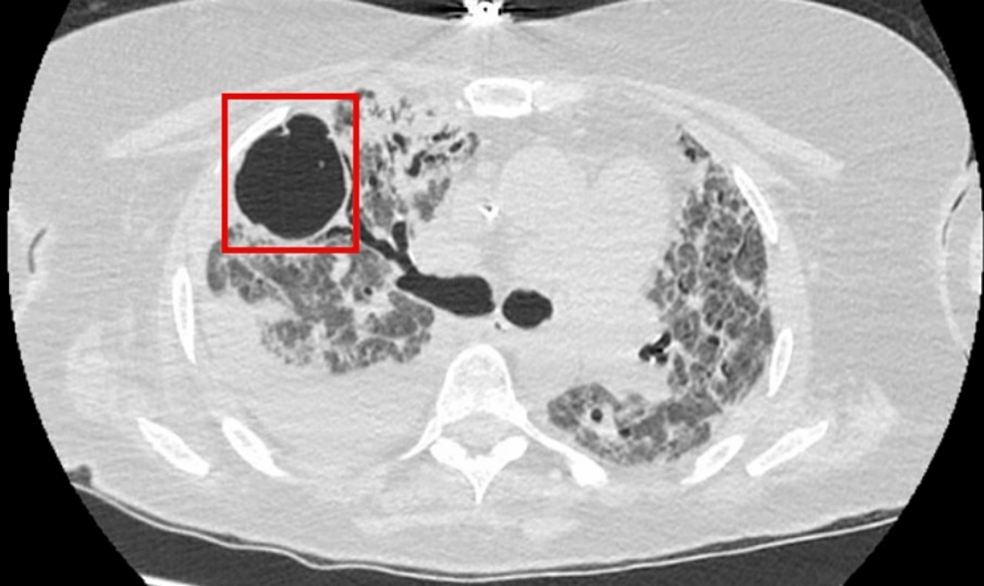
CT thorax showing 5 cm air-filled cyst (red box). Of note, right-sided pleural effusion continues to be present.

The patient made slow clinical and radiological progression in the following days. Her fevers had settled, and her ventilatory settings decreased. Steady-state voriconazole trough levels were observed (2.6 µg/mL). The patient could not tolerate a spontaneous breathing trial, given that she was intubated for 45 days. Per the family's wishes, the patient obtained a tracheostomy and percutaneous endoscopic gastrostomy (PEG) tube. A CT thorax was obtained on day 56 and showed diffuse bilateral interstitial and alveolar infiltrates throughout with a 5 cm air-filled cyst in the anterior right upper lobe (Figure 2).

The patient was discharged to a long-term acute care hospital with continuing ventilatory support. She was transitioned to PO voriconazole (400 mg q12) to finish her 12-week antifungal course. Despite the completion of treatment, the patient continues to require ventilatory support and remains in a long-term facility.

Case 2

We describe the case of a 54-year-old woman who presented with fever, chills, cough and gradually worsening shortness of breath. A month prior, she initially tested positive for COVID-19 at an urgent care clinic, where she presented for cough, chills, and postnasal drip. Her medical history was significant for type II diabetes mellitus with diabetic neuropathy, bipolar disorder, hyperlipidemia, and chronic low back pain. She was not vaccinated against COVID-19 and denied any exposure to sick contacts.

**Figure 3:**
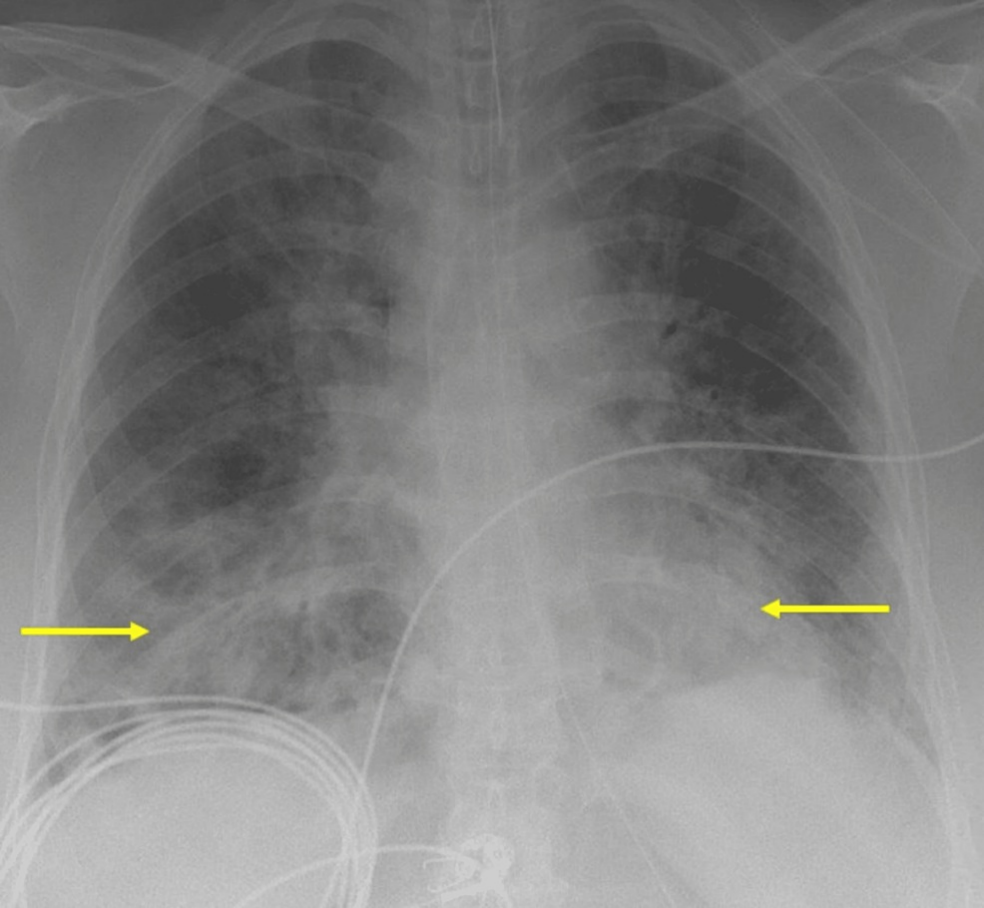
Chest X-ray demonstrating bilateral infiltrates suggestive of COVID-19 pneumonia (yellow arrows).

Upon presentation to the emergency department, she was febrile to 39°C with a pulse of 88/min, respiratory rate of 22/min with increased work of breathing and bilateral rhonchi, and oxygen saturation of 88%, which improved to 91% on 5 L/min oxygen via nasal cannula. The rest of the physical examination was normal. A nasopharyngeal swab was positive for SARS-CoV-2 RNA (RT-PCR method). Chest X-ray showed extensive bilateral lower lobe infiltrates consistent with COVID-19 pneumonia (Figure 3).

She was admitted to general medicine service and was started on remdesivir (200 mg IV once followed by 100 mg daily for 5 days), dexamethasone 6 mg IV daily, and a one-time infusion of Tocilizumab (IV 8mg/kg ). On day 4 of admission, the patient started expectorating thick, yellow-colored sputum and desaturated to 80% on minimal movement. She required up to 10 L/min of oxygen via an oxy mask. CT angiography thorax showed extensive bilateral airspace disease with ground-glass opacities, and diffuse infiltrates suggestive of COVID-19 pneumonia. A dense left lower lobe consolidation was suggestive of superimposed bacterial pneumonia. There was no evidence of a pulmonary embolism. She was started on IV Cefepime (2g q8h) and vancomycin (1250mg q24h) and was transferred to the ICU for increased oxygen requirements. BiPAP was trialed without a good response, and a decision was made to electively intubate the patient on day 6 of admission. Just before intubation, her P/F ratio was 78; after intubation and appropriate sedation, it improved to 97. Her respiratory status gradually declined over the next few days, with P/F ratios as low as 66 on lung-protective ventilation per ARDSnet protocol, indicating severe ARDS. She developed a left-sided spontaneous pneumothorax, and a chest tube was placed to water seal.

**Figure 4:**
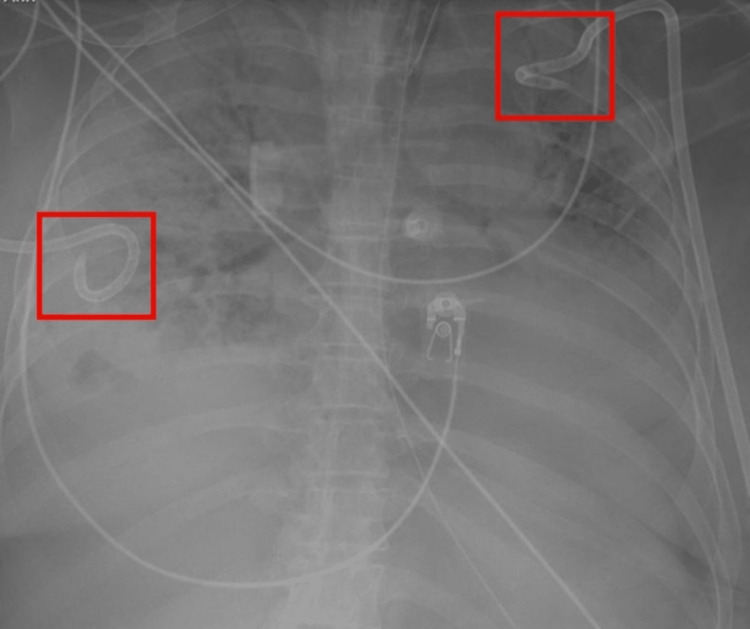
Chest X-ray demonstrating complete opacification of the patient's lungs. The red squares demonstrate the chest tubes.

The patient was evaluated by Extra-Corporeal Membrane Oxygenation (ECMO) service on day 10 of admission, and a decision was made to start Veno-venous (VV) ECMO. The cannulation procedure was complicated by post-procedural cardiac arrest due to hemorrhagic shock, and she was resuscitated with the return of spontaneous circulation after 8 minutes. Unfortunately, she developed a right-sided hemopneumothorax during bag-mask ventilation. A chest tube was inserted on the right side, and she was placed on lung rest ventilator settings while ECMO was being titrated to achieve adequate oxygenation. On Day 13 of admission, the patient started having low-grade fevers, and a chest X-ray showed near-total opacification of both lungs (Figure 4). Table 1 compares the patient's lab work from admission and Day13+.

**Table 1:** Case 2 lab work from admission (Day 0) and Day 13+ suggestive of an underlying infection of Aspergillus Fumigatus.

	Day 0	Day 13+
White Blood Count (WBC) [/µL]	6.1 x 10^3^	24 x 10^3^
C-Reactive Protein (CRP) [mg/L]	81	57
Procalcitonin [ng/mL]	0.16	0.63

Repeated blood cultures showed no bacterial or fungal colonies; however, serum Aspergillus galactomannan antigen assay was positive with an index of 1.26. A diagnostic bronchoscopy was done at the bedside on day 16 of admission, which showed no mucus plugging, but endobronchial washings grew multiple colonies of Aspergillus fumigatus. On Day 18, the patient was started on Liposomal Amphotericin B (IV 5 mg/kg) per the Department of Infectious Diseases recommendations. Despite aggressive medical management in the ICU, the patient passed away on day 21 from multi-organ sepsis.

Case 3 

A 54-year-old man with a past medical history of chronic kidney disease stage 4, hypertension, and type 2 diabetes presented to our ICU with worsening dyspnea 11 days after he initially tested positive for COVID-19. Before the presentation, the patient was experiencing fever, chills, shortness of breath, fatigue, and poor oral intake. Upon arrival, the patient was hypothermic with a temperature of 31.4°C, tachypneic with RR of 30, bradycardic with heart rate (HR) between 40 to 60, and oxygen saturation as low as 83%. The patient was subsequently intubated to maintain his oxygen saturation. His ventilator settings were pressure control(PC)/assist control (RR = 16, PEEP = 10, PC = 16). CT angiography thorax did not show any sign of a pulmonary embolism. However, he had bilateral ground glass opacities consistent with COVID-19 infection. He was started on IV dexamethasone (6 mg once daily), remdesivir (initial dose IV 200 mg; IV 100 mg q24 after that), and one dose of IV 400 mg sarilumab. The patient required emergent dialysis for potassium of 7 with electrocardiogram changes showing sinus bradycardia with mildly prolonged PR interval. He was placed on continuous venovenous hemofiltration (CVVH) after that. His sputum culture the following day grew Enterobacter, and he was started on meropenem.

On day 3, the sputum culture grew mold. We questioned the likelihood of a fungal infection given his early hospital course and short duration of steroids. The Department of Infectious Disease agreed that the validity of the mold in the sputum was questionable and treatment should be withheld. The Department of Infectious disease also recommended deescalating meropenem to IV levofloxacin which was dosed according to the patient's renal function (IV 750 mg q48 hours). The sputum culture had speciated to Enterobacter homacaei. On day 6, the patient started to decompensate, requiring norepinephrine to maintain his mean arterial pressure (MAP) above 60. Since the mold had speciated to Aspergillus fumigates, the decision was made to start IV voriconazole (6 mg/kg q12 for 2 doses, then 4 mg/kg q12 after that) and repeat a sputum culture. Initially, the repeat sputum culture taken on day 6 did not grow Aspergillus. Given the improvement in the patient's respiratory status to minimal ventilatory settings (RR = 14, PEEP = 8, PC = 12), it was decided to discontinue the voriconazole. The following day (Day+7), his sputum culture grew one colony of Aspergillus fumigatus. The patient completed a ten-day course of IV levofloxacin for his Enterobacter pneumonia.

**Figure 5:**
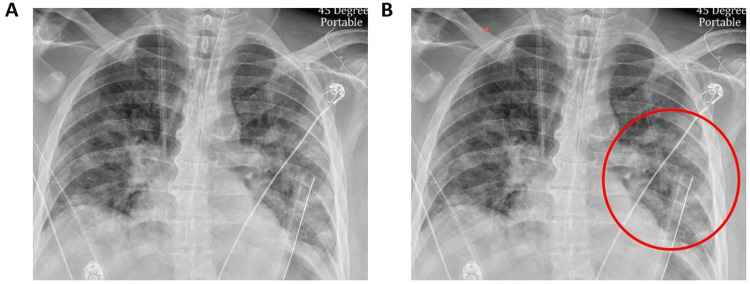
CXR prior to intubation (A) and after re-intubation (B). The red circle represents worsening infiltrates on the left side compared to the right.

The patient continued to be periodically hypothermic with a temperature of 35ºC and required pressor support. An extubation was attempted on day 14. A few hours later, his oxygen saturation had declined to 62%, requiring re-intubation. The patient was in refractory shock requiring 5 pressors and an elevated lactic acid of 8 that was up-trending. The source was thought to be a septic shock. However, there was no clear source of infection. Chest x-ray showed worsening bilateral infiltrates (Figure 5).

It was unclear whether the patient had aspirated or whether this was the worsening of his pulmonary infection with Aspergillus. He was placed on broad-spectrum antibiotics with vancomycin, piperacillin-tazobactam, and IV voriconazole to cover for the Aspergillus. The family had decided to pursue comfort care measures for the patient, given his poor prognosis. The patient passed away shortly after extubation on day 15.

## Discussion

COVID-19-associated pulmonary Aspergillosis (CAPA) is a secondary Aspergillus mold infection characterized by invasive pulmonary Aspergillosis (IPA) that occurs in COVID-19 patients [5]. These cases highlight the clinical characteristics, diagnostic criteria, and treatment of hospitalized CAPA patients. IPA has been seen after severe viral-related pneumonia, especially in patients admitted to the ICU for respiratory failure [6,7]. IPA has been reported in up to 15% of COVID-19 patients [2], which was histologically diagnosed in a series of 45 consecutive COVID-19 laboratory-confirmed autopsies [8].

Severe COVID-19 pneumonia destroys the bronchial mucosa creating favorable conditions for fungal growth [8]. The virus allows for vascular and epithelial permeability, facilitating the invasion of Aspergillus species [9]. Severe COVID-19 is associated with dysregulation of the immune system and overexpression of pro-and anti-inflammatory cytokines contributing to a highly permissive inflammatory environment enhancing fungal growth [10]. 

Clinical features associated with pulmonary Aspergillosis in patients with COVID-19 vary considerably. In one case series containing 20 patients, pulmonary Aspergillosis had been diagnosed a median of 11 days after symptom onset of COVID-19 and 9 days after intensive care unit admission [11]. This correlates with the third case we presented. The patient was not treated for CAPA due to the assumption of colonization rather than an actual infection, given his short ICU duration and course of steroids. However, based on the number of days of symptoms on admission (11 days), he might have had an actual CAPA infection resulting in his multi-organ sepsis and demise. The risk factors that have widely been reported include underlying factors like structural lung damage caused by COPD, immunosuppressant drugs, widespread use of broad-spectrum antibiotics, and HIV/AIDS, which tend to increase the risk for CAPA [2]. In addition, dexamethasone renders critically ill patients more susceptible to CAPA. Tocilizumab and Sarilumab, IL-6 inhibitors used in the treatment of COVID-19, inhibit the development of protective T-helper cells (Th17 cells), leading to an inadequate immune response [12]. 

The most common method to diagnose Aspergillus is to recover the fungus on culture media with bronchoalveolar lavage (BAL) or tracheal aspirate. Serologic biomarker testing, such as the conventional galactomannan (GM) from BAL, tracheal aspirate, and serum specimens, is also used to diagnose Aspergillus infection. Other diagnostic tests that may prove helpful also include PCR and serum (1→3)-β-d-glucan (BDG); however, these techniques are relatively less sensitive [13]. It is challenging to interpret positive cultures obtained from the respiratory tract as an actual infection or colonization. Distinguishing invasive Aspergillosis and COVID-19 pneumonia can be difficult from a radiological perspective, given that they share many similarities. For example, the "halo sign" seen in invasive Aspergillosis could be mistaken for a pulmonary infarction in a severe COVID-19 infection due to vascular injury via microthrombi [14,15]. Mechanically ventilated COVID-19 patients without invasive Aspergillosis often have nodular infiltrates, complicating the identification of surrounding halos in the scans [3].

An actual CAPA infection is proven with histopathological or cultural microscopic detection of the fungi showing invasive growth into the tissues [5]. Bronchoscopy is one of the few ways to obtain enough respiratory material for culture or histology. However, only some patients can undergo a bronchoscopy making the diagnosis of proven CAPA difficult. Probable CAPA should be suspected if there is Aspergillus from the BAL, positive serum GM index ≥0.5, positive BAL GM index of ≥1.0, or the presence of a new nodule or cavitary lesion(s) on chest CT [16]. The combination of multiple and repetitive positive mycology tests with typical radiological and clinical criteria contributes to the diagnosis of CAPA. Bronchoscopy should be the diagnostic gold standard whenever CAPA is suspected, including tracheobronchial inspection and BAL sampling for culture and GM [4].

Azoles with activity against Aspergillus species like Voriconazole or Isavuconazole are the first-line treatment options for CAPA [1]. Liposomal amphotericin B can be considered an alternative agent [1,17]. The most used antifungal agent is voriconazole due to its low cost and easy availability, followed by Isavuconazole and liposomal amphotericin [17]. Voriconazole is hepatically metabolized, and patients should be monitored for possible drug interactions with cytochrome P450 family 2 subfamily C member 19 (CYP2C19) and CYP3A [17]. Therapeutic drug monitoring should be performed as increased levels may lead to hepatotoxicity and neurotoxicity. Isavuconazole is associated with less severe adverse events and exhibits fewer drug-drug interactions, but it is costly. Isavuconazole should be preferred in patients for whom liver toxicity is a concern [17]. At the same time, the role of liposomal amphotericin could be limited by acute kidney injury complicating severe COVID-19 [1].

## Conclusions

COVID-19 predisposes the host to IPA as an independent host factor, as seen in the presented cases. IPA is recognized as an essential co-infection and cause of mortality in patients with severe COVID-19. The incidence will vary across ICUs. In settings where CAPA is known to occur commonly, screening for IPA in blood and true BAL samples (i.e., obtained via bronchoscopy) should be implemented, followed by preemptive treatment in those with mycological evidence of IPA. In other high-incidence settings, clinical antifungal prophylaxis trials should be considered among COVID-19 patients admitted to the ICU. The goal is to decrease the overall mortality of patients with CAPA using a multidisciplinary approach of infectious disease specialists, intensivists, pulmonologists, and clinical microbiologists.

## References

[1] Lai CC, Yu WL (2021). COVID-19 associated with pulmonary aspergillosis: a literature review. J Microbiol Immunol Infect.

[2] Janssen NA, Nyga R, Vanderbeke L (2021). Multinational observational cohort study of COVID-19-associated pulmonary aspergillosis. Emerg Infect Dis.

[3] White PL, Dhillon R, Cordey A (2021). A national strategy to diagnose coronavirus disease 2019-associated invasive fungal disease in the intensive care unit. Clin Infect Dis.

[4] Armstrong-James D, Youngs J, Bicanic T (2020). Confronting and mitigating the risk of COVID-19 associated pulmonary aspergillosis. Eur Respir J.

[5] Chong WH, Saha BK, Neu KP (2022). Comparing the clinical characteristics and outcomes of COVID-19-associate pulmonary aspergillosis (CAPA): a systematic review and meta-analysis. Infection.

[6] Schauwvlieghe AF, Rijnders BJ, Philips N (2018). Invasive aspergillosis in patients admitted to the intensive care unit with severe influenza: a retrospective cohort study. Lancet Respir Med.

[7] Magira EE, Chemaly RF, Jiang Y, Tarrand J, Kontoyiannis DP (2019). Outcomes in invasive pulmonary aspergillosis infections complicated by respiratory viral infections in patients with hematologic malignancies: a case-control study. Open Forum Infect Dis.

[8] Fortarezza F, Boscolo A, Pezzuto F (2021). Proven COVID-19-associated pulmonary aspergillosis in patients with severe respiratory failure. Mycoses.

[9] Bradley BT, Maioli H, Johnston R (2020). Histopathology and ultrastructural findings of fatal COVID-19 infections in Washington State: a case series. Lancet.

[10] Hoenigl M (2021). Invasive fungal disease complicating coronavirus disease 2019: when It rains, it spores. Clin Infect Dis.

[11] Marr KA, Platt A, Tornheim JA, Zhang SX, Datta K, Cardozo C, Garcia-Vidal C (2021). Aspergillosis complicating severe coronavirus disease. Emerg Infect Dis.

[12] Honda H, Kida H, Yoshida M (2011). Recurrent allergic bronchopulmonary aspergillosis in a patient with rheumatoid arthritis treated with etanercept and tocilizumab. Mod Rheumatol.

[13] Arastehfar A, Carvalho A, van de Veerdonk FL (2020). COVID-19 associated pulmonary aspergillosis (CAPA)-from immunology to treatment. J Fungi (Basel).

[14] Aquino SL, Kee ST, Warnock ML, Gamsu G (1994). Pulmonary aspergillosis: imaging findings with pathologic correlation. AJR Am J Roentgenol.

[15] Kanne JP, Bai H, Bernheim A (2021). COVID-19 imaging: what we know now and what remains unknown. Radiology.

[16] Zhang SX, Balada-Llasat JM, Pancholi P, Sullivan KV, Riedel S (2021). COVID-associated pulmonary aspergillosis in the United States: is it rare or have we missed the diagnosis?. J Clin Microbiol.

[17] Feys S, Almyroudi MP, Braspenning R, Lagrou K, Spriet I, Dimopoulos G, Wauters J (2021). A visual and comprehensive review on COVID-19-associated pulmonary aspergillosis (CAPA). J Fungi (Basel).

